# Quantifying the roles of visual, linguistic, and visual-linguistic complexity in noun and verb acquisition

**DOI:** 10.1371/journal.pone.0321973

**Published:** 2025-05-23

**Authors:** Yuchen Zhou, Michael J. Tarr, Daniel Yurovsky

**Affiliations:** 1 Department of Psychology, Carnegie Mellon University, Pittsburgh, PA, United States; 2 Neuroscience Institute, Carnegie Mellon University, Pittsburgh, PA, United States; 3 Machine Learning Department, Carnegie Mellon University, Pittsburgh, PA, United States; UCSD: University of California San Diego, UNITED STATES OF AMERICA

## Abstract

Children often learn the meanings of nouns before they grasp the meanings of verbs. This discrepancy could arise from differences in the complexity of visual characteristics for categories that language describes, the inherent structure of language, or how these two sources of information align. To explore this question, we analyze visual and linguistic representations derived from large-scale pre-trained artificial neural networks of common nouns and verbs, focusing on these three hypotheses about early verb learning. Our findings reveal that verb representations are more variable and less distinct within their domain compared to nouns. When only one example per category is available, the alignment between visual and linguistic representations is weaker for verbs than for nouns. However, with multiple examples (mirroring human language development), this alignment improves significantly for verbs, approaching that of nouns. This suggests that the difficulty in learning verbs is not primarily due to mapping visual events to verb meanings, but rather in forming accurate representations of each verb category. Regression analysis indicates that visual variability significantly impacts verb learning, followed by the alignment of visual and linguistic elements and linguistic variability. Our study provides a quantitative and integrative framework to account for the challenges children face in early word learning, opening new avenues for resolving the longstanding debate on why verbs are harder to learn than nouns.

## Introduction

Children’s early vocabularies are dominated by nouns [1]. Although there is some variability in the size of the effect across cultures and languages, children learn nouns more quickly than verbs and other predicates [2, 3]. Efforts to understand the complexities involved in learning verbs have typically concentrated on identifying if the main obstacles arise from the organization of categories in the real world to which language refers, the intrinsic structure of the language, or the alignment between these two elements.

To know the meaning of a word, learners must learn the perceptual category to which each word refers [4, 5]. Consequently, much of the work on early word learning is concerned with the cognitive processes children use to map words to visual categories [6–8]. In this context, one explanation for the challenges of verb learning focuses on the relative difficulty of visual category learning: referents labeled by verbs may be more variable or more confusable than those labeled by nouns [9, 10]. This difference arises because nouns typically correspond to objects that are naturally consistent and individuated, as proposed by the Natural Partitions Hypothesis [9, 11, 12]. Objects in the world tend to form discrete, concrete entities that are easier to recognize and categorize. For example, “dogs” differ from each other on a variety of dimensions, such as color, size, and fur texture, but share a common basic shape [13]. In contrast, “walks” differ from each other not only in dimensions such as speed, direction, number of limbs, and gait but also in the identity of the agent doing the walking.

A second explanation for the challenges of verb learning focuses on the linguistic contexts formed by other words in which a target word appears. Along the lines of Firth’s intuition that “you shall know a word by the company it keeps” [14], congenitally blind children can correctly name the colors of common objects and even the similarities among these colors, even though color is a purely visual property [15]. In this context, the difficulty of verb learning may be attributable, at least in part, to the fact that verbs refer to relations among nouns and thus are likely to occur in more diverse linguistic contexts. Supporting this account, children learn verbs that occur in more restricted contexts earlier than verbs that occur in more diverse contexts [16].

Finally, the difficulty of learning verbs may emerge from the alignment between visual and linguistic sources of information, a phenomenon explained by the Relational Relativity Hypothesis [9]. Under this view, verbs are more difficult to learn than nouns because their linguistic usage is less transparently related to the events with which they occur. For example, English tends to encode the manner of the motion into the verb, but not the direction (e.g., as in “The bottle floated into the cave.”), while Spanish tends to lexicalize the direction of the movement when describing the same event, leaving manner as an optional adjunct (e.g., as in “La botella entró en la cueva, flotando.”, meaning “The bottle entered the cave floating.”) [9]. Furthermore, the contexts of transitive verbs are fundamentally ambiguous unless the words for their agent and patient are known (e.g., as in “chase” and “flee”) [17]. Accordingly, verbs may require selecting among the available relationships and identifying the syntactic structure of the utterances.

Tests of these explanations have typically relied on cross-linguistic comparisons [18], studies of atypical learners [19, 20], or artificial language learning experiments that try to approximate the real-world learning problems faced by children [21]. Here, we introduce a novel method for quantifying the complexity of both the visual and linguistic contexts in which a word appears, as well as the alignment of both sources of information. By independently assessing these different sources of information for word learning, we can directly assess their explanatory value in addressing the challenges associated with learning verbs. Specifically, we examined how the complexity and alignment of visual and linguistic information impact the learning of common nouns and verbs, selecting these words based on their frequency of occurrence.

## Measuring visual and linguistic complexity

To better understand how visual information, linguistic information, and visual/linguistic alignment contribute to differences in the learnability of nouns and verbs, it is critical to represent both sources of information in a unified manner. Historically, top-down, hand-designed approaches have predominated in the psychological study of visual meaning structures, where experimenters have explicitly defined and manipulated features such as color, size, and shape [22, 23]. In contrast, due to the arbitrary relationship between the form of a word and its meaning [24], a bottom-up approach has been adopted in the study of the structure of individual word meanings, where word meanings are assumed to emerge from the regularity of word statistics. Modern language models build on similar intuitions regarding word co-occurrences, but with more powerful learning algorithms and larger and more diverse training corpora. While these language models such as BERT and recent “Large Language Models” do not explicitly encode predefined meanings [25–29], these models produce representations that align with human similarity judgments [30], predict human behavior in semantic fluency tasks [31], predict choices in classical decision-making tasks [32], and capture top-down linguistic features identified by linguists, such as syntax [33]. Therefore, we propose that embeddings derived from these language models serve as a reasonable approximation of meaning and leverage these advances to explore the underlying structure of word meanings.

In addition to generating word embeddings from the language model [27], we similarly use a pre-trained vision model to generate visual embeddings from pixels that represent the visual structure of the categories to which words refer [34]. Importantly, we intentionally chose a vision model trained via an unsupervised learning method to ensure that the model encodes only visual features, such as color, texture, and edges.

## Learning verbs versus learning nouns

Given a unified way to represent the linguistic and visual dimensions of word meanings as vectors, we then apply common measures to examine whether the later onset of verb learning arises from one or more of the following factors: 1) identifying stable visual categories; 2) understanding meanings from linguistic contexts; or 3) aligning the visual and linguistic structures corresponding to the same word.

**Fig 1:**
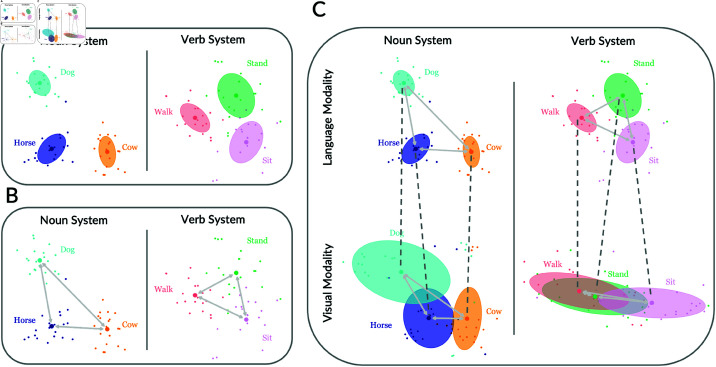
Potential sources of difficulty for learning nouns and verbs as illustrated by concepts in embedding spaces. Large dots represent category centroids, small dots represent exemplars of corresponding categories, and colored ellipses represent 68% confidence (one standard deviation) areas for each category (see Methods for visualization details). (A) Within each category, verb exemplars are more variable as compared to noun exemplars; (B) Verb categories overlap more with one another and are less distinguishable from one another as compared to noun categories; (C) Within the verb system, the structures of linguistic and visual representations are inconsistent across modalities, while in the noun system, the structures of linguistic representations and visual representations are better aligned.

Intuitively, the meaning of a word may be hard to learn from the contexts in which it appears for two different reasons. First, different instances of a word may carry different meanings. This variability makes it difficult to converge on the central meaning of a word. Second, two different words may appear in similar contexts, making the words confusable. One method for quantifying these two sources of difficulty is to consider both the similarities between multiple instances of the same word and the dissimilarities between different words. For example, as illustrated in Fig 1A, the distribution of verb exemplars may be more scattered compared to noun exemplars. At the same time, as illustrated in Fig 1B, verb categories may generally be less similar to one another as compared to noun categories.

We can also measure, as illustrated in Fig 1C, the alignment between the linguistic representation of a word and the visual representation of the corresponding category. Prior work has demonstrated that representations across these two modalities tend to be highly aligned for concrete nouns, and, furthermore, that the earliest learned words are more highly aligned than later learned words [35]. Here, we explore whether the degree of alignment between the linguistic and visual meanings of words helps distinguish noun learning from verb learning. We hypothesize that nouns will have a visual structure more aligned with their linguistic structure, whereas the visual and linguistic structures of verbs will be less aligned.

Finally, although each of these measures may independently predict some of the difficulty of word learning, we are interested in the main obstacles that children face when acquiring the meaning of words. Accordingly, we estimate the relative contributions of each of these measures in a single regression analysis to determine how well each factor can predict the age at which a given word is learned.

## Results

As a first step, we examine the category structure in each modality using neural network-based representations (see Methods for details) of 210 nouns and 210 verbs from the Visual Genome (VG) dataset [36]. The complete list of words is provided in S2 Appendix. We use this word dataset to investigate the difficulty faced by a simulated language learner in forming new categories and differentiating between separable categories. Under this rubric, the category structure is captured by two measures: 1) the degree of dispersal for each individual category (“variability”); and 2) how far categories are from each other (“discriminability”). Accordingly, these structural measures of representational space are quantified by two metrics: the variability of individual categories and the discriminability of different categories (see Methods for mathematical definitions).

### Category structure in the visual modality

**Fig 2:**
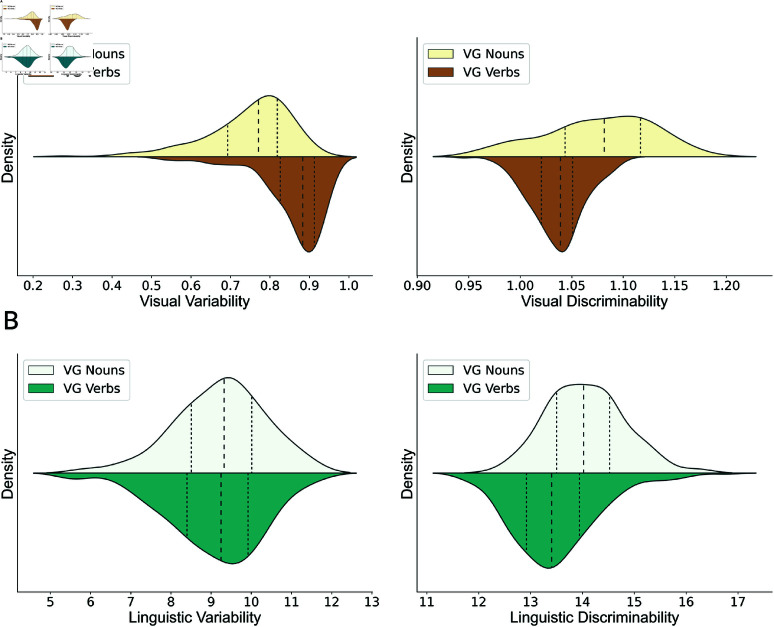
Distribution of variability and discriminability of nouns and verbs from the Visual Genome (VG) dataset. Dashed lines denote 25th-, 50th-, and 75th-percentile of distributions. (A) Visual modality: Verb categories are more variable and less distinguishable than noun categories. (B) Language modality: Verb categories are equally variable but less distinguishable than noun categories.

The visual variability of verbs is significantly greater than that of nouns, t(418)=11.71, *p* < 0.001, and the visual discriminability of verbs is significantly lower than that of nouns, t(418)=−9.82, *p* < 0.001 (Fig 2A). Consistent with our hypothesis, objects labeled by the same noun are more like one another than events labeled by the same verb, and objects described by different nouns are less confusable with one another than events described by different verbs.

### Category structure in the language modality

Although the difference in linguistic variability is not statistically significant between nouns and verbs, t(418)=−1.15, *p* = 0.250, the linguistic discriminability of verbs is reliably lower than the linguistic discriminability of nouns, t(418)=−7.63, *p* < 0.001 (Fig 2B). These results provide evidence that the linguistic meanings of verbs are less distinguishable compared to those of nouns.

In sum, the observations from unimodal visual and linguistic analyses offer an explanation for why verbs are harder to learn than nouns: Compared to nouns, it is harder to capture the central meanings of the visual and linguistic categories of verbs and to distinguish between two visual categories of verbs.

### Alignment between visual and language modalities

Another challenge in learning verbs is that they filter and highlight specific relational information from physical events. This requires learners to determine which physically grounded details are captured within different verb categories. To explore this possibility, we examine the crossmodal alignment between the visual and language modalities of nouns and verbs, asking whether visual categories can be readily mapped onto linguistic categories. We term this measure “Alignment Strength” (see Methods for mathematical definition). To make well-controlled comparisons across systems with different words, we also measure the relative strength of alignment, that is, how well aligned a mapping is compared to alternative permuted mappings in which linguistic and visual representations of different words are randomly matched.

**Fig 3:**
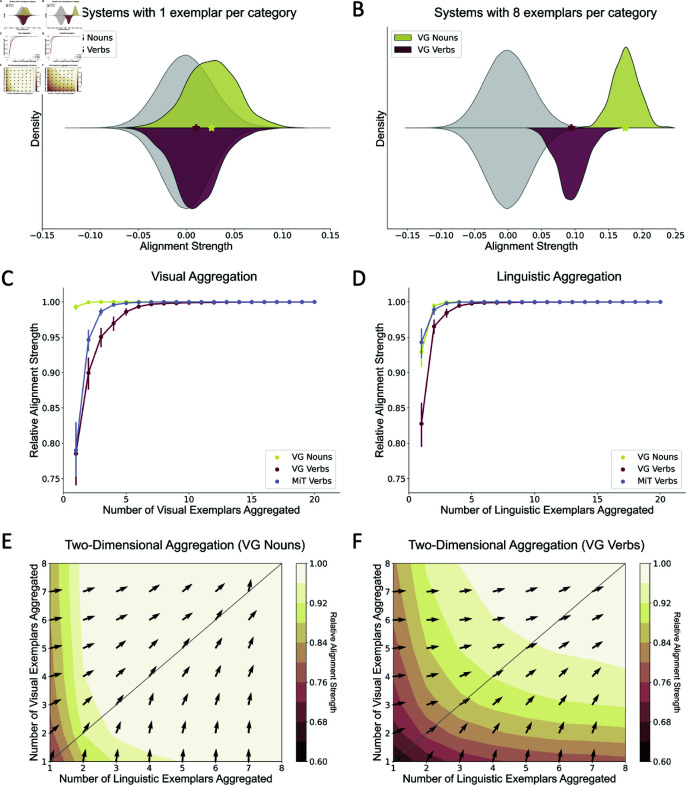
Alignment between visual and language modalities. (A) The distribution of alignment strengths of true mappings (colors) and randomly permuted mappings (grey) with one linguistic and one visual exemplar per category. Nouns are more well-aligned than verbs. (B) The distribution of permuted mappings (grey) in systems with 8 linguistic exemplars and 8 visual exemplars aggregated per category. The alignment strength improves considerably as compared to that in the single-exemplar condition (as in A) for both nouns and verbs. Asterisks represent the mean alignment strengths of the true mappings in both (A) and (B). As visual exemplars (C) or linguistic exemplars (D) aggregate when the other modality has sufficient exemplars to form well-learned prototypes, the relative alignment strength, which measures the percentage of permuted mappings that are less well-aligned than the true mapping, increases and gradually converges to 1.0. The noun system is more well-aligned than the verb system in early aggregation stages, but the verb system becomes almost as well-aligned as the noun system with a sufficient number of learning instances. Error bars represent 95% confidence intervals computed by bootstrapping over 1,000 simulations at each level. (E, F) Relative alignment strength as a function of both the number of linguistic and visual exemplars. Nouns can become highly aligned with only one visual exemplar and sufficient linguistic exemplars per category. In contrast, sufficient visual exemplars and sufficient linguistic exemplars are both necessary for the formation of a well-aligned verb system.

Fig 3A shows the distribution of alignment strengths of true mappings and permuted mappings in systems with one visual exemplar representing each visual category and one linguistic exemplar representing each linguistic category. The mean alignment strength of true mappings is significantly different from the mean of permuted mappings for both nouns, t(1,000,998)=30.35, *p* < 0.001, and verbs, t(1,000,998)=11.17, *p* < 0.001, suggesting that true mappings are reliably more aligned than randomly permuted mappings.

The mean alignment strength of true mappings for nouns (ρ=0.027) is higher than 75.6% of permuted mappings, while the mean alignment strength of true mappings for verbs (ρ=0.009) is higher than 59.8% of permuted mappings. Consistent with our hypothesis, as captured by both absolute alignment strength and relative alignment strength, it is more difficult to establish correct mappings for verbs compared to nouns. Thus, identifying the correct mapping between visual categories and linguistic categories may be more challenging in verb learning than in noun learning.

### Improving alignment by exemplar aggregation

An alternative explanation for the lower alignment strength observed for verbs could be the high variability and low discriminability of verb categories. As such, the specific meaning of a verb may not be well represented by a single exemplar. Consistent with how humans gradually acquire word meanings over multiple exemplars, we aggregate exemplars by taking their arithmetic mean to examine the change in alignment strength as a function of the number of exemplars.

For visual aggregation, we form well-learned stable linguistic prototypes by aggregating 20 linguistic exemplars per category. We then calculate the relative alignment strength of the system as a function of how many visual exemplars are provided (Fig 3C). For nouns, the observed true mapping has a higher alignment strength than nearly all of the permuted mappings even with just one visual exemplar per category; additional exemplars produce little improvement in the alignment strength. In contrast, the alignment strength is quite low for verbs with one visual exemplar per category; however, additional exemplars produce a progressive increase in the alignment strength. Indeed, at the point at which eight visual exemplars per category are aggregated, the alignment strength of the verb system approaches that of the noun system.

Mirroring visual aggregation, with well-learned visual prototypes formed by 20 exemplars per category provided, the alignment strengths of both nouns and verbs improve as linguistic exemplars are aggregated (Fig 3D). However, in contrast to visual aggregation, for linguistic aggregation, we find that one linguistic exemplar per category cannot produce a good mapping for the noun system. Rather, three or more linguistic exemplars per category are needed to build a well aligned noun mapping. This difference in the number of required exemplars between visual aggregation and linguistic aggregation is consistent with empirical observations from previous studies showing that 3- and 4-month-old infants can discriminate two basic-level visual categories (i.e., cats and horses) after 3 exposures to each stimulus image [37], yet 3- and 4-year-old children cannot understand the meanings of novel words very well after 12 exposures to the words in storybooks [38]. For verbs, similar to our findings for visual aggregation, the alignment strength is quite low with one linguistic exemplar per category; however, additional exemplars produce a progressive increase in the alignment strength.

Two-dimensional aggregation simulations, where both visual and linguistic exemplars are aggregated, reveal the optimal combination of visual and linguistic exemplars that most efficiently increase alignment strength at different learning stages (Fig 3E and 3F). For example, suppose at a given point the gradient points 45 degrees counterclockwise from the x-axis. In that case, it suggests an optimal 1:1 ratio between visual and linguistic exemplars, meaning the learner needs both visual and linguistic input equally. Conversely, if the gradient points close to 90 degrees, it indicates that visual exemplars are more impactful, and adding linguistic exemplars has minimal effect on further increasing alignment strength. We found that verb systems demonstrate a quasi-symmetric pattern (e.g., the alignment strength of the verb system with one visual exemplar and eight linguistic exemplars is close to that of eight visual exemplars and one linguistic exemplar), whereas the noun system displays a strong bias towards linguistic input (e.g., the alignment strength of the noun system with one visual exemplar and eight linguistic exemplars is much better than that of the noun system with eight visual exemplars and one linguistic exemplar). This result is consistent with the **natural partitions hypothesis** [9, 11], which posits that concrete nouns are naturally individuated referents and therefore noun learning relies less on learning corresponding visual categories.

To summarize, aggregating multiple exemplars improves the alignment strength for both nouns and verbs. Importantly, consistent with the success of children learners over time, with sufficient exemplars, the visual and linguistic meanings of verbs can be transparently mapped. This finding contradicts our initial hypothesis, suggesting that there is no inherent global misalignment between visual events and verb usage. The reason is that if such a misalignment existed, it would not be possible to raise the alignment strength to an almost perfect level, no matter how many exemplars are provided. Therefore, we conclude that the difficulty in verb learning largely lies in the challenges of extracting invariant categorical representations of verbs rather than any misalignment between visual events and verb usage. Supporting this conclusion, a replication using a second image dataset yields similar qualitative results (refer to S1 Appendix). These findings are illustrated in S1 Fig, S2 Fig, and S3 Fig. The complete list of words is provided in S3 Appendix.

### Action-based verb representation

Nouns typically denote objects that are stable physical entities. In contrast, verbs typically denote actions that unfold dynamically over time and space [39]. Consequently, verb representations derived from static images of actions may miss important aspects of verb learning. As an illustrative example, single images of “stand” and “walk” are visually similar, but the two actions are easily distinguishable from each other in videos. However, it is also possible that a few “key frames” depicting the most diagnostic information may be sufficient to learn the meaning of a verb. Videos may be less efficient than images because they contain irrelevant background objects and actions that may make learning more challenging. To explore these questions, we replicate our analyses using 305 action words from a video dataset, Moments in Time [40], and examine whether the dynamic nature of verbs impacts the alignment of the verb system. The complete list of words is provided in S4 Appendix.

Representations derived from videos of actions (MiT) exhibit a similar alignment pattern to representations derived from static images (VG Verbs): the alignment strength is low at first, but improves incrementally and ultimately hits the ceiling after multiple linguistic/visual exemplars are aggregated (S2 Fig). Notably, consistent with the idea that action dynamics are helpful in verb learning, the number of exemplars per category required for good mapping using videos is fewer compared to the number required using static images. This suggests that videos do carry dynamic information that is relevant to verb meaning and therefore support verb learning better than static images.

### Unpacking the contributions of different factors

Our analyses demonstrate that multiple factors, including variability, discriminability, and alignment strength, reflect important characteristics of word learning. Furthermore, a wide variety of studies have documented that word frequency is a significant predictor of when a word will be learned [41–45]. To answer the ultimate question of what drives early word learning, we use a gradient-boosted trees model, “XGBoost” [46], to run a regression analysis that takes into account all of these factors, as well as word frequency and word type to predict the learnability of words. As a proxy for the learnability of words, Age of Acquisition (AoA) is measured using CHILDES [47], a child-directed speech corpus. Following previous literature [42], AoA is defined as the age at which 50% of children in the corpus demonstrate an understanding of a given word.

**Fig 4:**
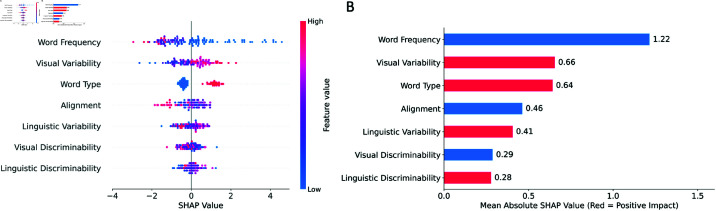
Visualization of feature contributions in word learning. An XGBoost model was trained to predict the learnability of words as measured by Age of Acquisition (AoA). SHAP (SHapley Additive exPlanations) [48] values were computed to interpret the relative contribution of each feature. (A) Summary of the importance of all features in predicting AoA. Each dot represents one word category. If the SHAP value of a category with regard to a particular feature is positive, then this feature positively impacts the predicted variable in this observation and vice versa. For instance, frequency negatively influences AoA because high word frequency generally corresponds to a low SHAP value (“type” refers to word type with verbs encoded as 1 and nouns encoded as 0). (B) Global feature importance quantified by the mean absolute SHAP values of each feature across all word categories. Features that are positively correlated with the predicted variable (AoA) are colored red, while features that are negatively correlated with AoA are colored blue.

For better interpretability of results, we adopt SHAP (SHapley Additive exPlanations) [48] method to visualize the predictions made by the model. Fig 4A illustrates the contribution of each factor in predicting AoA. Each dot corresponds to one word category, and the position of the dot regarding 0 on the x-axis reflects whether this factor positively or negatively contributes to the value of AoA. Consistent with previous studies, we find that frequency is a reliable predictor of AoA: words with high frequencies typically have low AoAs. Word type is also distinctly associated with the learnability of words: without other information, knowing a word is a verb always increases the expected AoA of the word.

Because the contributions of other factors are less obvious visually, we compute the global importance of each factor by calculating the mean of absolute SHAP values of each factor across all words (Fig 4B). All factors, with the exception of linguistic discriminability, are correlated with AoA as hypothesized: word frequency, alignment, and visual discriminability positively impact AoA, while visual variability, word type, and linguistic variability negatively impact AoA. Interestingly, visual variability is such a strong predictor of AoA that it is comparable to word type in magnitude. In contrast, both visual and linguistic discriminability show only a small contribution in predicting AoA, which may explain the incorrect prediction of our model with respect to the impact of linguistic discriminability.

Compared to the effects of factors with established benchmarks (word frequency and word type), visual variability appears to be the most important among the additional factors we considered. At the same time, alignment and linguistic variability are also reliably associated with the difficulty of learning words, while neither visual discriminability nor linguistic discriminability predicts when a word will be learned.

## Discussion

A long-standing question in the study of language development is why verbs are harder to learn than nouns. In this paper, we examine the relative contribution of three sources of information: (1) the structure of visual categories in the world to which language refers; (2) the structure of language itself; and (3) the interplay between visual and linguistic information. Supporting the natural partitions hypothesis, which states that nouns associated with concrete objects are easy to acquire because they are naturally consistent and individuated referents [9, 11, 12], we demonstrate that for nouns: (1) robust mappings between nouns and corresponding visual objects can be established with just one encounter with the visual object; (2) robust mappings between nouns and corresponding visual objects can be established with a relatively small number of encounters with linguistic instances for each noun; (3) grasping word meanings, rather than visual object recognition, is the main obstacle to overcome in noun learning. In contrast, verbs associated with dynamic events are held to be harder to acquire because they are more variable. That is, the actions themselves and the identity of the agents performing the actions and the patients receiving the actions vary across different contexts. Supporting this hypothesis, an examination of embedding spaces demonstrates that verbs are more visually variable than nouns. More generally, we find that all three factors discussed above contribute to the difficulty of word learning, but visual variability is the most predictive of the age at which children learn individual words. This finding is consistent with earlier observations that more imaginable concepts (evoking mental images more easily) are acquired more rapidly and earlier in development [12, 39].

Another theoretical point is that verbs are hard to learn because they make a selection of both particular objects in the environment and the relationships between those objects. This is different from nouns, which involve a direct mapping between words and unitary visual objects. Reflecting this difference between verbs and nouns, there is evidence that the selection of particular properties of actions emphasized by verbs varies across languages while nouns are relatively consistent cross-linguistically [9, 11, 18]. One implication of this theory is that children need to put more effort into identifying the core properties of verb categories as they learn a language (e.g., attending more to the structure of visual examples of verbs and to the larger context of linguistic examples). However, our results suggest that increased attention or other forms of effort to identify core properties of verbs are not necessarily a difficulty to the effective acquisition of verb meanings. We demonstrate that mapping referents with verbs can be resolved by exemplar aggregation, suggesting that the main difficulty in learning verbs lies in the challenge of extracting invariant categorical representations of verbs rather than any inherent global misalignment between visual events and verb usage. In other words, the way by which the English linguistic space is carved up is not arbitrary and is, in fact, structured similarly to how events visually appear to be. To better understand what kinds of relational information are selected to lexicalize events and how that may affect language learning, future studies should pursue cross-linguistic comparisons. It is also possible that alignment strength as a global measurement is not sensitive to local distortions (children may confuse “push” with “pull” but are less likely to confuse “push” with “run”), so more fine-grained analyses would be helpful in addressing this question.

Our analyses leverage neural network derived representations to simulate how a learner might encounter noun and verb exemplars in real life and extract key information. Although the physical architectures and training objectives of human brains and neural networks clearly differ, both must efficiently capture statistical patterns from sensory inputs to support downstream tasks. As a result, they produce representations that, while potentially differing in form, often encode similar underlying information. Some recent evidence indicates that models performing better on downstream tasks exhibit representations more closely aligned with human neural activity [49, 50]. It is also important to note that each neural network, constrained by its architecture, training algorithm, and the dataset used for training, instantiates only one way of representing an image or a word. These underlying assumptions can introduce biases into subsequent analyses. However, models with a greater number of parameters and more diverse training data may help to mitigate these limitations, as they are less likely to be biased towards a specific subset of stimuli and their representations encode more comprehensive and nuanced information.

Our present work stands in contrast to previous studies on early language acquisition that generally relied on a limited number of case studies or in-lab experiments where stimuli were designed and manipulated manually. Instead, we sample learning exemplars of a relatively large number of words from large corpora and use artificial neural networks that were trained in an unsupervised fashion to allow representations to emerge bottom-up. Most importantly, by encoding visual and linguistic information in the same representational form, our quantitative analyses provide an integrative view, rather than an all-or-nothing mindset, on the difficulties that children face in acquiring nouns and verbs. As such, our approach opens a new door for resolving the long-lasting debate on why verbs are harder to learn than nouns.

The mechanisms underlying children’s rapid word learning are undoubtedly more complex than what we have considered in our analyses. For example, our main focus is on word-level semantic learning and does not take into account other documented factors, including inflectional morphology [51], syntactic constructions [52], and the ease with which a word can be segmented from a continuous speech stream [53]. Moreover, in our approach, children are treated as passive learners in that we only examine language comprehension; factors such as interactions between children and parents/caregivers [54, 55] and children’s intentions [56, 57] are also likely to play a role in guiding language learning. In addition, while our work effectively explains the general trend of why nouns are typically learned earlier than verbs, it does not account for finer-grained effects, such as certain verbs being learned earlier than some nouns or how early-learned nouns and verbs may facilitate the acquisition of other words [58]. However, the primary contribution of our study lies not only in our results, but in presenting a methodological framework for exploring early language acquisition. This framework is flexible and can be readily applied to investigate specific subsets of words. For instance, it could be used to compare early- and late-learned words or to analyze how the first acquired words contribute to the learning of subsequent vocabulary. We believe this approach is an important step forward and has the potential to model a wide range of phenomena in language development and broad concept learning.

## Materials and methods

### Datasets and models

Visual Genome (VG) was used as the dataset to sample visual images of nouns and verbs [36]. Images in VG have dense annotations of objects and relationships between objects in the form of Wordnet Synset [59], which lemmatizes synonymous concepts. We selected the 210 verbs that appear no less than 20 times in the whole dataset to form the verb system. Because nouns are generally more frequent than verbs, we selected the most frequent 210 nouns to ensure the size of the noun system is the same as the size of the verb system. The region of interest (ROI) of a noun is determined by the bounding box of the annotated object, and the ROI of a verb is determined by the union of the bounding boxes of any agents and/or objects that take part in the verb.

The video dataset we used is Moments in Time (MiT), which includes a collection of one million three-second video clips [40]. Each video clip features only one action and was annotated with one of 305 action words. Considering the length of videos, the visual representation of each video was generated by taking the arithmetic mean of the visual representations of 8 frames uniformly sampled from the video.

Visual representations were derived from a neural network based on ResNet-50 architecture [60]. The network was pre-trained in an unsupervised fashion by an algorithm called Swapping Assignments between Views on the ImageNet ILSVRC2012 dataset [34]. The unsupervised training paradigm ensures that the model was trained only to encode low-level visual features (e.g., colors, dots, edges).

For language data, we sampled texts that contain the 210 nouns/verbs from Wikipedia articles. We defined the context of a word to be the 25 words that precede the target word and the 25 that follow it, which sums up to a window of 51 words.

Bidirectional Encoder Representations from Transformers (BERT) [27] was employed to extract language representations of words because of its ability to produce contextual-sensitive embeddings as the contexts of the target word change. We obtained the pre-trained uncased BERT model from the Transformers library [61]. We used the hidden states of the last layer before the language modeling head as the embedding of the target word.

### Visualization in schematic diagram

We used representations from real data to illustrate our hypotheses. For each category, 20 exemplars were sampled and t-SNE was applied to project representations to a two-dimensional plane. Data were assumed to follow 2D Gaussian distributions for the estimation of parameters of ellipses. Embeddings in the same modality were plotted on the same scale for visual comparison. Linguistic representations from BERT were taken as an example for demonstration in panel A and panel B.

### Variability and discriminability

We quantified the structure of embedding spaces by two metrics: the variability of individual categories, which is computed as the average Euclidean distance from exemplars to category centroids, and the discriminability of different categories, which is computed as the average Euclidean distance from all exemplars in each category to category centroids of all other categories. Mathematically, given a system of *N* words, $W=\{w_{1},w_{2},\cdots ,w_{N}\}$, with *P* linguistic exemplars $L=\{l_{11},\cdots ,l_{1P},l_{21},\cdots ,l_{2P},\cdots ,l_{NP}\}$ and *Q* visual exemplars $V=\{v_{11},\cdots ,v_{1Q},v_{21},\cdots ,v_{2Q},\cdots ,v_{NQ}\}$ for each category, linguistic category centroids $LC=\{lc_{1},\cdots ,lc_{N}\}$ and visual category centroids $VC=\{vc_{1},\cdots ,vc_{N}\}$ are computed as


$$lc_{i}=\frac{1}{P}\sum_{j=1}^{P}l_{ij},\;vc_{i}=\frac{1}{Q}\sum_{j=1}^{Q}v_{ij}$$

Linguistic variability and visual variability are then defined as


$$lv_{i}=\frac{1}{P}\sum_{j=1}^{P}dist(l_{ij},lc_{i}),$$

$$vv_{i}=\frac{1}{Q}\sum_{j=1}^{Q}dist(v_{ij},vc_{i})$$

while linguistic and visual discriminability are defined as


$$ld_{i}=\frac{1}{(N-1)P}\underset{j\neq i}{\sum_{j=1}^{N}}\sum_{k=1}^{P}dist(l_{jk},lc_{i}),$$

$$vd_{i}=\frac{1}{(N-1)Q}\underset{j\neq i}{\sum_{j=1}^{N}}\sum_{k=1}^{Q}dist(v_{jk},vc_{i})$$

where *dist* computes the Euclidean distance between two points in an embedding space.

### Alignment strength

To estimate the alignment between words’ linguistic and visual representations, we used a metric termed “Alignment Strength”, which asks whether words’ linguistic similarities are correlated with their visual similarities. Computationally, we first constructed the similarity matrices of representations within the visual modality, $S_{V}$, and language modality, *S*_*L*_, respectively by computing the pairwise cosine similarity of representations. The alignment strength was then computed as Spearman’s rank correlation between the upper triangle of $S_{V}$ and *S*_*L*_.

The relative alignment strength is defined as the percentage of misaligned mappings that have lower alignment strength than the true mapping system. For each true mapping, we randomly sampled 1,000 mappings that were misaligned (e.g., the visual “dog” mapped to language “cat” while the visual “cat” mapped to language “dog”), and then computed the alignment strength of those misaligned systems as a reference for the true mapping.

We randomly sampled 1,000 systems to estimate the alignment strength of systems with 1 visual exemplar and 1 linguistic exemplar per category (Fig 3A). Similarly, we randomly sampled 1,000 systems to estimate the alignment strength of systems with 8 visual exemplars and 8 linguistic exemplars per category (Fig 3B).

### Exemplar aggregation

Exemplar aggregation was achieved by taking the arithmetic mean of exemplars from the same category. For visual aggregation, we first created stable linguistic prototypes from 20 linguistic exemplars per category (*post-hoc* experiments confirmed that 20 exemplars per category can form well-learned linguistic prototypes). Instead of forming systems by purely random sampling, we incrementally added one visual exemplar to the existing system and compared the alignment strength of the true system with 1,000 permuted mappings to simulate the learning process of humans. We ran 1,000 simulations for each level of number of visual exemplars. The manipulation of linguistic aggregation was identical to visual aggregation, where the roles of visual modality and language modality were exchanged.

The procedure for two-dimensional aggregation was also similar, where 500 simulations were run for each possible combination of number of visual exemplars and number of linguistic exemplars.

### Regression analysis

AoAs were measured directly from children’s utterances in child-directed speech by vocabulary data retrieved from the Wordbank database [62]. Specifically, AoA is defined as the age at which 50% of children in the corpus demonstrate understanding of a given word, following previous literature [42]. Estimates of the frequency of words that children hear were based on parental/caregivers’ speech transcripts from the CHILDES database [47].

Instead of conventional linear models, we chose a type of gradient-boosted tree model called XGBoost [46] for the regression analysis because tree-based models make fewer assumptions about the relationship between input and output and are immune to the issue of multi-collinearity, which is especially important when a large number of predictor variables are involved. The XGBoost model was trained for 10,000 runs with a max depth of 10 and a learning rate of 0.02.

## Acknowledgments

We thank Leila Wehbe, Brian MacWhinney, Erik Thiessen, and Graham Neubig for their helpful comments.

## Supporting information

S1 Appendix Replication on VRD dataset. (PDF)

S1 Fig Distribution of variability and discriminability of nouns and verbs from the Visual Relationship Detection (VRD) dataset. (PDF)

S2 Fig Relative alignment strength as a function of the number of visual/linguistic exemplars per category on the Visual Genome (VG), Visual Relationship Detection (VRD), and Moments in Time (MiT) datasets. (PDF)

S3 Fig Relative alignment strength as a function of both the number of linguistic exemplars and the number of visual exemplars on the Visual Relationship Detection (VRD) dataset. (PDF)

S2 Appendix Words in the Visual Genome dataset. (PDF)

S3 Appendix Words in the Visual Relationship Detection dataset. (PDF)

S4 Appendix Words in the Moments in Time dataset. (PDF)

## References

[1] Bates E, Marchman V, Thal D, Fenson L, Dale P, Reznick JS, et al. Developmental and stylistic variation in the composition of early vocabulary. J Child Lang. 1994;21(1):85–123. doi: 10.1017/s0305000900008680 8006096

[2] Fenson L, Dale PS, Reznick JS, Bates E, Thal DJ, Pethick SJ, et al. Variability in early communicative development. Monogr Soc Res Child Dev. 1994;59(5):i–185. doi: 10.2307/1166093 7845413

[3] Frank MC, Braginsky M, Yurovsky D, Marchman VA. Variability and consistency in early language learning: the Wordbank project. MIT Press; 2021.

[4] Peirce CS. Collected papers of Charles Sanders Peirce. Harvard University Press; 1974.

[5] Quine W, Van O. Word and object: an inquiry into the linguistic mechanisms of objective reference. John Wiley; 1960.

[6] Landau B, Smith LB, Jones SS. The importance of shape in early lexical learning. Cogn Dev. 1988;3(3):299–321. doi: 10.1016/0885-2014(88)90014-7

[7] Markman EM, Wachtel GF. Children’s use of mutual exclusivity to constrain the meanings of words. Cogn Psychol. 1988;20(2):121–57. doi: 10.1016/0010-0285(88)90017-5 3365937

[8] Waxman SR, Markow DB. Words as invitations to form categories: evidence from 12-to 13-month-old infants. Cognitive Psychol. 1995;29(3):257–302. 10.1006/cogp.1995.1016 8556847

[9] Gentner D. Why nouns are learned before verbs: linguistic relativity versus natural partitioning. Center for the Study of Reading Technical Report No 257; 1982.

[10] Golinkoff RM, Hirsh-Pasek K. How toddlers begin to learn verbs. Trends Cogn Sci. 2008;12(10):397–403. doi: 10.1016/j.tics.2008.07.003 18760656

[11] Gentner D. Some interesting differences between verbs and nouns. Cogn Brain Theory. 1981;4(2):161–78.

[12] Gentner D, Boroditsky L, Bowerman M, Levinson S. Individuation, relativity, and early word. Lang Cult Cogn. 2001;3:215–56.

[13] Rosch E, Mervis CB, Gray WD, Johnson DM, Boyes-Braem P. Basic objects in natural categories. Cogn Psychol. 1976;8:382–439.

[14] Firth JR. A synopsis of linguistic theory, 1930–1955. Stud Linguist Anal; 1957.

[15] Landau B, Gleitman LR, Landau B. Language and experience: evidence from the blind child, vol. 8. Harvard University Press; 2009.

[16] Goldberg AE, Casenhiser DM, Sethuraman N. Learning argument structure generalizations. Cogn Linguist. 2004.

[17] Gleitman L. The structural sources of verb meanings. Lang Acquisit. 1990;1(1):3–55. doi: 10.1207/s15327817la0101_2

[18] Talmy L. Semantics and syntax of motion. In: Syntax and semantics volume 4. Brill; 1975. p. 181–238.

[19] Snedeker J, Geren J, Shafto CL. Starting over: international adoption as a natural experiment in language development. Psychol Sci. 2007;18(1):79–87. doi: 10.1111/j.1467-9280.2007.01852.x 17362382

[20] Goldin-Meadow S, Feldman H. The development of language-like communication without a language model. Science. 1977;197(4301):401–3. doi: 10.1126/science.877567 877567

[21] Gillette J, Gleitman H, Gleitman L, Lederer A. Human simulations of vocabulary learning. Cognition. 1999;73(2):135–76. doi: 10.1016/s0010-0277(99)00036-0 10580161

[22] Shepard RN, Hovland CI, Jenkins HM. Learning and memorization of classifications. Psychol Monogr: Gen Appl. 1961;75(13):1–42. doi: 10.1037/h0093825

[23] Ashby FG, Maddox WT. Human category learning. Annu Rev Psychol. 2005;56(1):149–78. doi: 10.1146/annurev.psych.56.091103.070217 15709932

[24] De Saussure F. Cours de linguistique g´en´erale, vol. 1. Otto Harrassowitz Verlag; 1989.

[25] Mikolov T, Chen K, Corrado G, Dean J. Efficient estimation of word representations in vector space. arXiv. 2013.

[26] Pennington J, Socher R, Manning CD. Glove: global vectors for word representation. In: Proceedings of the 2014 conference on empirical methods in natural language processing (EMNLP). 2014. p. 1532–43.

[27] Devlin J, Chang MW, Lee K, Toutanova K. Bert: pre-training of deep bidirectional transformers for language understanding. arXiv. 2018.

[28] Brown T, Mann B, Ryder N, Subbiah M, Kaplan JD, Dhariwal P, et al. Language models are few-shot learners. Adv Neural Inform Process Syst. 2020;33:1877–901.

[29] Ouyang L, Wu J, Jiang X, Almeida D, Wainwright CL, Mishkin P, et al. Training language models to follow instructions with human feedback. arXiv. 2022.

[30] Hill F, Reichart R, Korhonen A. Simlex-999: evaluating semantic models with (genuine) similarity estimation. Comput Linguist. 2015;41(4):665–695. doi: 10.1162/coli_a_00237

[31] Hills TT, Jones MN, Todd PM. Optimal foraging in semantic memory. Psychol Rev. 2012;119(2):431. doi: 10.1037/a0027373 22329683

[32] Bhatia S. Associative judgment and vector space semantics. Psychol Rev. 2017;124(1):1. doi: 10.1037/rev0000047 28004958

[33] Hu J, Gauthier J, Qian P, Wilcox E, Levy RP. A systematic assessment of syntactic generalization in neural language models. arXiv preprint arxiv. 2020.

[34] Caron M, Misra I, Mairal J, Goyal P, Bojanowski P, Joulin A. Unsupervised learning of visual features by contrasting cluster assignments. Adv Neural Inform Process Syst. 2020;33:9912–24.

[35] Roads BD, Love BC. Learning as the unsupervised alignment of conceptual systems. Nat Mach Intell. 2020;2(1):76–82. doi: 10.1038/s42256-019-0132-2

[36] Krishna R, Zhu Y, Groth O, Johnson J, Hata K, Kravitz J, et al. Visual genome: connecting language and vision using crowdsourced dense image annotations. 2016. Available from: https://arxiv.org/abs/1602.07332

[37] Eimas PD, Quinn PC. Studies on the formation of perceptually based basic-level categories in young infants. Child Dev. 1994;65(3):903–17. doi: 10.1111/j.1467-8624.1994.tb00792.x 8045176

[38] Horst JS, Parsons KL, Bryan NM. Get the story straight: contextual repetition promotes word learning from storybooks. Front Psychol. 2011;2:17. doi: 10.3389/fpsyg.2011.00017 21713179 PMC3111254

[39] McDonough C, Song L, Hirsh-Pasek K, Golinkoff RM, Lannon R. An image is worth a thousand words: why nouns tend to dominate verbs in early word learning. Dev Sci. 2011;14(2):181–9. doi: 10.1111/j.1467-7687.2010.00968.x 21359165 PMC3043374

[40] Monfort M, Andonian A, Zhou B, Ramakrishnan K, Bargal SA, Yan T, et al. Moments in time dataset: one million videos for event understanding. IEEE Trans Pattern Anal Mach Intell. 2019;42(2):502–8. doi: 10.1109/TPAMI.2019.2901464 30802849

[41] Consortium M. Quantifying sources of variability in infancy research using the infant-directed-speech preference. Adv Methods Pract Psychol Sci. 2020;3(1):24–52.

[42] Goodman JC, Dale PS, Li P. Does frequency count? Parental input and the acquisition of vocabulary. J Child Lang. 2008;35(3):515–31. 18588713 10.1017/S0305000907008641

[43] Ebbinghaus H. Memory: A contribution to experimental psychology. Ann Neurosci. 2013;20(4):155. doi: 10.5214/ans.0972.7531.200408 25206041 PMC4117135

[44] Brown GD, Watson FL. First in, first out: word learning age and spoken word frequency as predictors of word familiarity and word naming latency. Mem Cognit. 1987;15(3):208–216. doi: 10.3758/bf03197718 3600260

[45] Ambridge B, Kidd E, Rowland CF, Theakston AL. The ubiquity of frequency effects in first language acquisition. J Child Lang. 2015;42(2):239–73. 25644408 10.1017/S030500091400049X PMC4531466

[46] Chen T, Guestrin C. Xgboost: A scalable tree boosting system. In: Proceedings of the 22nd ACM SIGKDD International conference on knowledge discovery and data mining. 2016. p. 785–94.

[47] MacWhinney B. The CHILDES project: tools for analyzing talk, volume II: the database. Psychology Press; 2014.

[48] Lundberg SM, Lee SI. A unified approach to interpreting model predictions. Adv Neural Inform Process Syst. 2017;30.

[49] Schrimpf M, Blank IA, Tuckute G, Kauf C, Hosseini EA, Kanwisher N, et al. The neural architecture of language: integrative modeling converges on predictive processing. Proc Natl Acad Sci U S A. 2021;118(45):e2105646118. doi: 10.1073/pnas.2105646118 34737231 PMC8694052

[50] Conwell C, Prince JS, Kay KN, Alvarez GA, Konkle T. A large-scale examination of inductive biases shaping high-level visual representation in brains and machines. Nat Commun. 2024;15(1):9383. doi: 10.1038/s41467-024-53147-y 39477923 PMC11526138

[51] Brown R. A first language. Harvard University Press; 2013.

[52] Ninio A. Pathbreaking verbs in syntactic development and the question of prototypical transitivity. J Child Lang. 1999;26(3):619–53. doi: 10.1017/s0305000999003931 10603698

[53] Saffran JR, Aslin RN, Newport EL. Statistical learning by 8-month-old infants. Science. 1996;274(5294):1926–8. doi: 10.1126/science.274.5294.1926 8943209

[54] Nelson K. Young minds in social worlds: experience, meaning, and memory. Harvard University Press; 2007.

[55] Adamson LB. Communication development during infancy. Routledge; 2018.

[56] Meltzoff AN. Understanding the intentions of others: re-enactment of intended acts by 18-month-old children. Dev Psychol. 1995;31(5):838. 25147406 10.1037/0012-1649.31.5.838 PMC4137788

[57] Gergely G, Nádasdy Z, Csibra G, Bíró S. Taking the intentional stance at 12 months of age. Cognition. 1995;56(2):165–93. doi: 10.1016/0010-0277(95)00661-h 7554793

[58] Longobardi E, Spataro P, Putnick DL, Bornstein MH. Do early noun and verb production predict later verb and noun production? Theoretical implications. J Child Lang. 2017;44(2):480–95. 26880050 10.1017/S0305000916000064 PMC5822724

[59] Miller GA. WordNet: a lexical database for English. Communi ACM. 1995;38(11):39–41. doi: 10.1145/219717.219748

[60] He K, Zhang X, Ren S, Sun J. Deep residual learning for image recognition. In: Proceedings of the IEEE conference on computer vision and pattern recognition. 2016. p. 770–8.

[61] Wolf T, Debut L, Sanh V, Chaumond J, Delangue C, Moi A, et al. Huggingface’s transformers: State-of-the-art natural language processing. arXiv. 2019:191003771.

[62] Frank MC, Braginsky M, Yurovsky D, Marchman VA. Wordbank: An open repository for developmental vocabulary data. J Child Lang. 2017;44(3):677–94. doi: 10.1017/S0305000916000209 27189114

