# Supplementary material for: Quantifying the roles of visual, linguistic, and visual-linguistic complexity in noun and verb acquisition
Source: PLoS One. 2025 May 23;20(5):e0321973. doi: 10.1371/journal.pone.0321973 (PMC12101840; doi:10.1371/journal.pone.0321973)
Supplement: S1 Appendix — (PDF) [file pone.0321973.s001.pdf]

S1 Appendix

Replication on VRD Dataset

To enhance the reliability of the study, we replicated our analyses on the Visual Relationship Detection dataset (VRD) [1]. This dataset consists of 5,000 real-world images, each with manually annotated bounding boxes for 100 object categories and 70 categories of predicate relationships between objects. We selected 53 verbs that appeared at least 10 times in the entire dataset to form the verb system and the most frequently occurring 53 nouns as the noun system. Similar to the pipeline of processing the Visual Genome dataset (the dataset we used in the main text), ResNet-SwAV and BERT were used to extract visual and linguistic representations respectively.

Category Structure

Visual Modality As shown in Figure S1, we found that the structure of the visual embedding space of VRD is qualitatively similar to that of VG. The visual variability of VRD verbs is significantly greater than that of VRD nouns, $t(104) = 6.44$, $p < 0.001$, and the visual discriminability of VRD verbs is significantly lower than that of VRD nouns, $t(104) = -10.38$, $p < 0.001$.

Language Modality The difference in linguistic variability is not statistically significant between VRD nouns and VRD verbs, $t(104) = 0.06$, $p = 0.954$, while the linguistic discriminability of VRD verbs is reliably lower than the linguistic discriminability of VRD nouns, $t(104) = -6.12$, $p < 0.001$.

Summary The visual structure and linguistic structure of VRD words are qualitatively similar to that of VG words. Compared to nouns, it is more challenging to capture the core meanings of verb categories, both visually and linguistically, and to differentiate between the visual categories of two verbs.

Exemplar Aggregation

To confirm whether the visual and linguistic representations of words in VRD can become well-aligned through aggregating exemplars, we conducted visual and linguistic aggregation separately. As illustrated in Figure S2, we observed a similar pattern to that seen for VG exemplar aggregation: aggregating exemplars enhances the alignment strength of both VRD nouns and verbs. In the early stages of aggregation, the noun system tends to be more easily aligned than the verb system. However, with an increasing number of learning instances, the verb system becomes almost as aligned as the noun system. This result is consistent with our finding from the VG dataset that the difficulty in learning verbs is mainly due to the challenges of extracting invariant categorical representations of verbs rather than any inherent global misalignment between visual events and word usage.

We also performed a two-dimensional aggregation to evaluate the comparative importance of acquiring new visual and linguistic exemplars at different learning stages. As depicted in Figure S3, the verb system exhibits a quasi-symmetric pattern such that the relative alignment strength of a verb system with M visual exemplars and N linguistic exemplars per category is roughly equivalent to that of a verb system with N visual exemplars and M linguistic exemplars per category. However, unlike VG nouns, the VRD noun system displays a preference for visual input: Even with numerous linguistic exemplars, the relative alignment strength of the noun system cannot approximate 100% without sufficient visual exemplars per category. Nevertheless, the

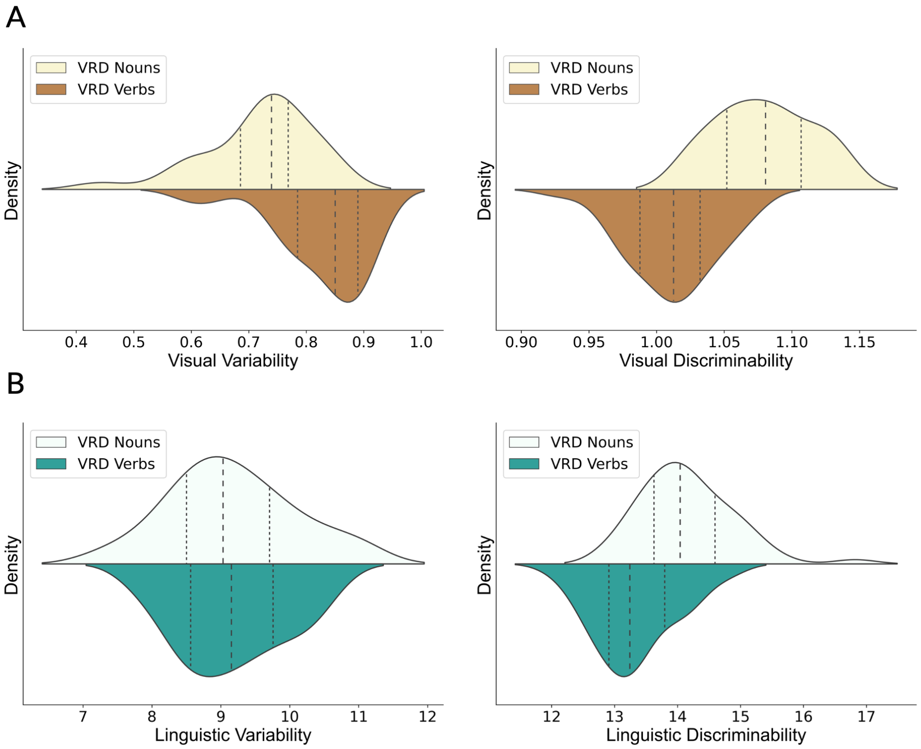

Fig S1. Distribution of variability and discriminability of nouns and verbs from the Visual Relationship Detection (VRD) Dataset. Dashed lines denote 25th-, 50th-, and 75th-percentile of distributions. (A) Visual modality: Verb categories are more variable and less distinguishable than noun categories. (B) Language modality: Verb categories are equally variable but less distinguishable than noun categories.

noun system can become well-aligned with just one linguistic exemplar per category with adequate visual exemplars. This implies that the challenge of learning nouns in the VRD dataset stems from extracting visual prototypes rather than comprehending the linguistic meanings of words. One possible explanation to account for this phenomenon is that each noun in VRD appears in a distinct linguistic context, making it less difficult to differentiate between nouns by their linguistic meanings. It is also possible that the sample size is too small to obtain a precise estimate of the actual effect.

Upon comparison across the three datasets (VG, MiT, and VRD), we demonstrate that nouns exhibit a higher degree of alignment than verbs during the early aggregation phases. Nevertheless, verbs reach a comparable level of alignment to nouns when provided with an adequate quantity of learning exemplars. Moreover, video-based exemplars prove to be more informative than those derived from static images.

References

1. Lu C, Krishna R, Bernstein M, Fei-Fei L. Visual relationship detection with language priors. In: Computer Vision–ECCV 2016: 14th European Conference, Amsterdam, The Netherlands, October 11–14, 2016, Proceedings, Part I 14. Springer; 2016. p. 852–869.

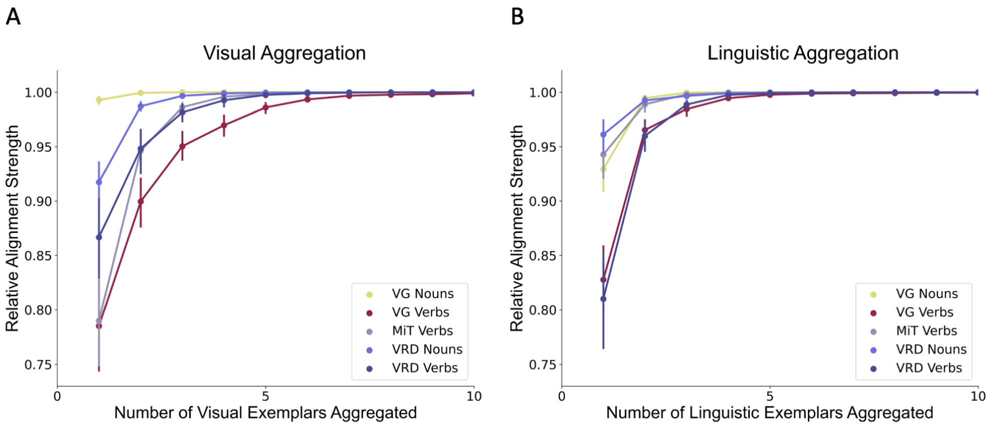

Fig S2. Relative alignment strength as a function of the number of visual/linguistic exemplars per category on the Visual Genome (VG), Visual Relationship Detection (VRD), and Moments in Time (MiT) datasets. As visual exemplars (A) or linguistic exemplars (B) aggregate, relative alignment strength increases and gradually converges to 1.0. Nouns are generally more well-aligned than verbs in early aggregation stages, but verbs become almost as aligned as nouns with a sufficient number of learning exemplars. Exemplars from the video dataset (MiT) are more informative than exemplars from image datasets. Error bars represent 95% confidence intervals computed by bootstrapping over 1,000 simulations at each level.

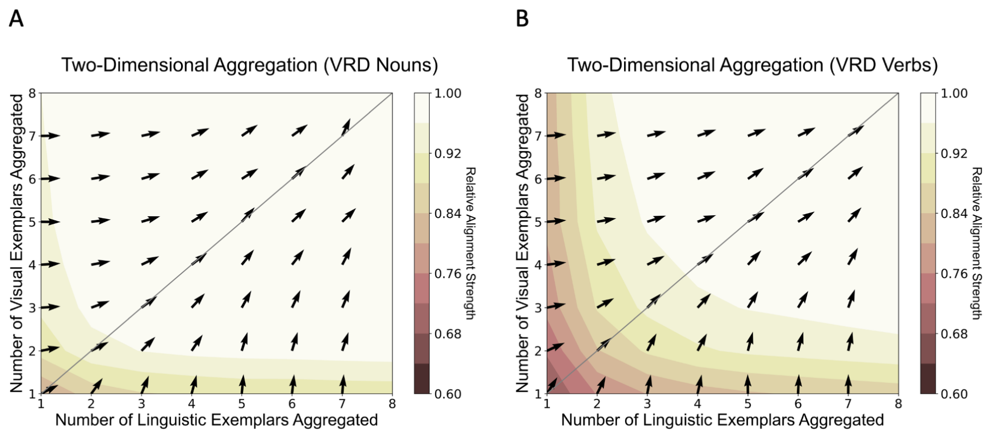

Fig S3. Relative alignment strength as a function of both the number of linguistic exemplars and the number of visual exemplars on the Visual Relationship Detection (VRD) dataset. (A) The noun system becomes well-aligned with just one linguistic exemplar per category with adequate visual exemplars. (B) In contrast, sufficient visual exemplars and sufficient linguistic exemplars are both necessary for the formation of a well-aligned verb system. Directions of arrows indicate directions of gradients, which represent the optimal combination of visual exemplars and linguistic exemplars that increases alignment strength most efficiently at each location.
