# Supplementary figures and images for: Quantifying the roles of visual, linguistic, and visual-linguistic complexity in noun and verb acquisition
Source: PLoS One. 2025 May 23;20(5):e0321973. doi: 10.1371/journal.pone.0321973 (PMC12101840; doi:10.1371/journal.pone.0321973)

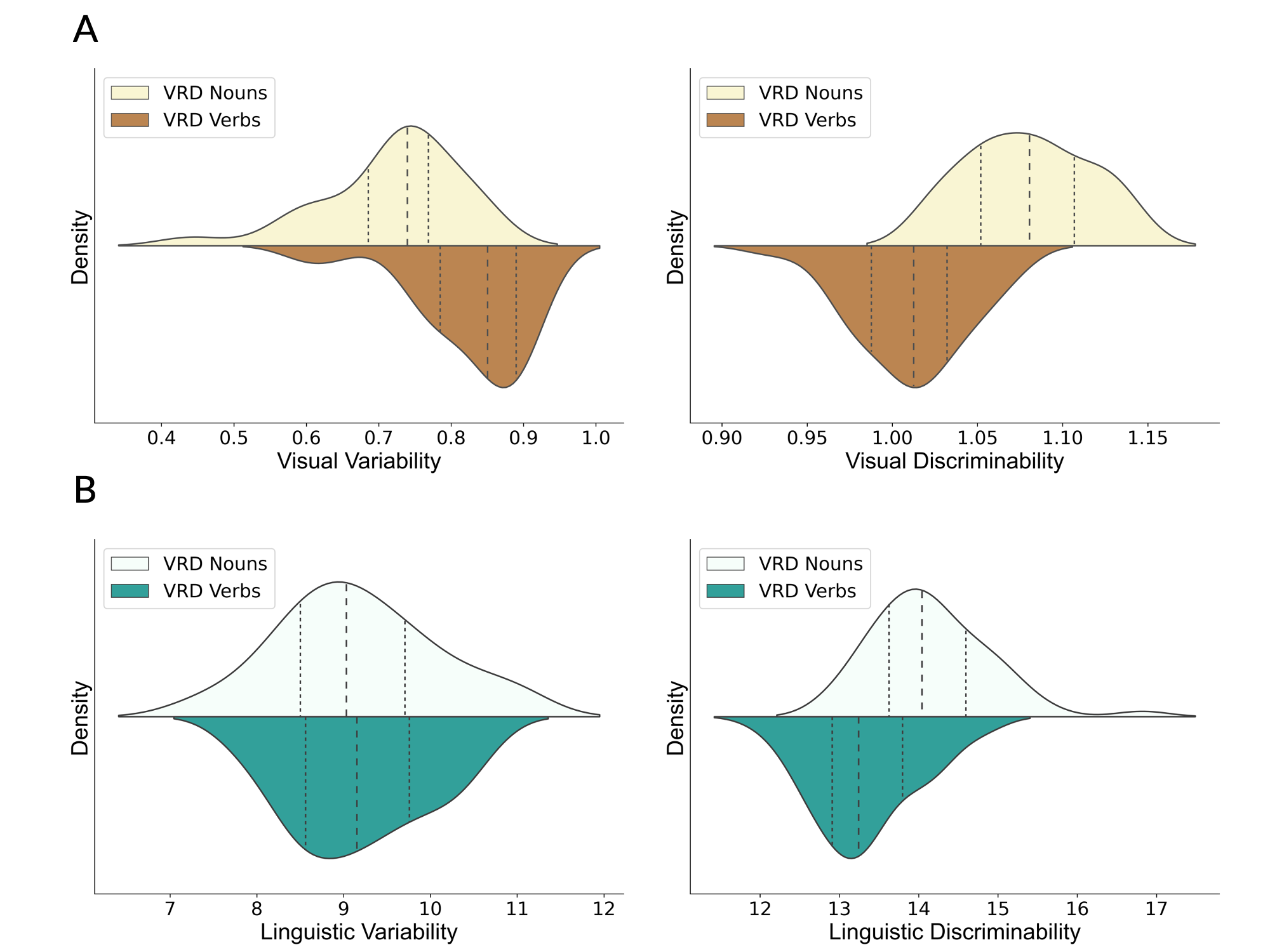

Supplement: S1 Fig — (PDF) [file pone.0321973.s002.tif]

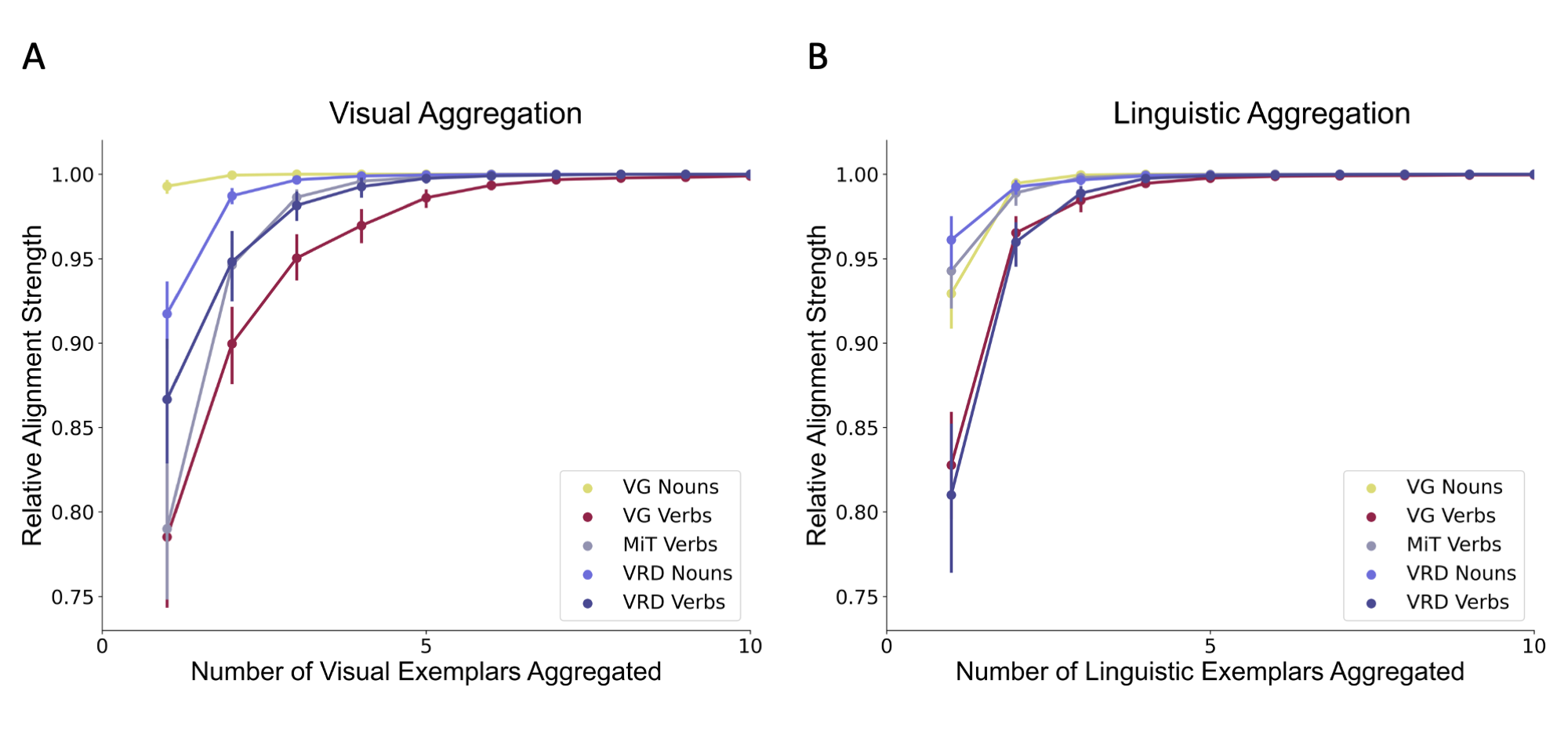

Supplement: S2 Fig — (PDF) [file pone.0321973.s003.tif]

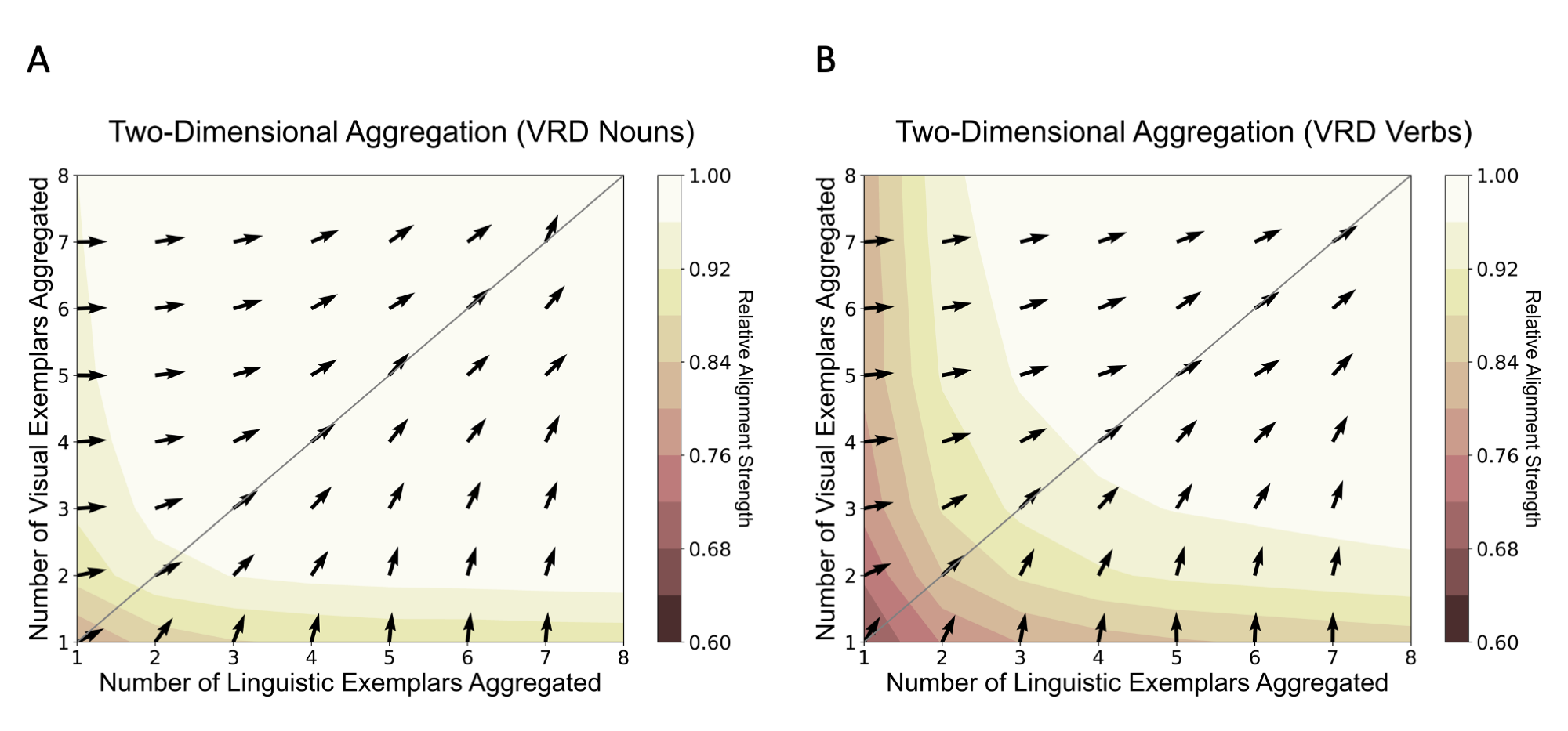

Supplement: S3 Fig — (PDF) [file pone.0321973.s004.tif]
