# Supplementary material for: Quantifying the roles of visual, linguistic, and visual-linguistic complexity in noun and verb acquisition
Source: PLoS One. 2025 May 23;20(5):e0321973. doi: 10.1371/journal.pone.0321973 (PMC12101840; doi:10.1371/journal.pone.0321973)
Supplement: S2 Appendix — (PDF) [file pone.0321973.s005.pdf]

S2 Appendix

Words in the Visual Genome Dataset

Nouns man, window, woman, building, person, tree, wall, shirt, sky, water, grass, train, airplane, hand, car, cloud, pole, dog, girl, bus, road, giraffe, snow, elephant, head, street, cat, people, field, horse, light, leaf, boat, leg, clock, zebra, fence, hair, bird, pizza, floor, bicycle, hat, ear, bench, door, line, sidewalk, bear, wheel, shadow, rock, eye, flower, child, bed, beach, shoe, cow, trouser, chair, jacket, motorcycle, skateboard, truck, bowl, surfboard, sheep, numeral, bag, tail, desk, mirror, food, shelf, glass, umbrella, spectacles, foot, player, face, nose, wave, letter, handle, bottle, counter, arm, animal, sand, lady, box, picture, vase, pillow, sink, banana, guy, house, book, shrub, ocean, toilet, tile, sofa, tire, hill, jean, mountain, laptop, logo, cup, plant, cap, paper, telephone, frisbee, ball, tower, post, wing, ceiling, button, ski, branch, trunk, roof, headlight, sunglasses, coat, sandwich, mouth, brick, flag, lamp, cabinet, necktie, court, neck, skier, fork, bathroom, railing, keyboard, writing, room, television, basket, tray, knife, seat, wire, suit, engine, towel, rug, container, bridge, baby, finger, air, cheese, pot, computer, vehicle, horn, apple, surfer, broccoli, stove, batter, watch, design, windshield, backpack, collar, fruit, bread, catcher, sock, photograph, teddy, word, dish, curtain, distance, kitchen, frame, blanket, napkin, paw, pavement, hole, dress, platform, ballplayer, wood, oven, back, camera, tag, boot, snowboard, park, river, carrot, jet, wrist, uniform, barroom.

Verbs have, be, wear, sit, stand, traverse, walk, attach, hang, put, play, eat, fly, watch, make, lie, use, tend, show, leave, state, swing, run, arrive, front, reach, pull, surround, fill, stay, reduce, park, associate, become, see, lead, float, wait, necessitate, talk, drink, drive, construct, read, write, close, travel, push, end, jump, tie, decorate, feed, climb, achieve, form, whiten, open, divide, work, discontinue, describe, enjoy, blacken, complain, serve, neglect, bend, glitter, chase, descend, pet, happen, punch, keep, match, light, board, protect, grip, try, sell, brown, hide, grok, meet, bite, solve, sniff, sleep, enter, gaze, approach, help, perform, give, herd, haul, smile, crash, visualize, load, wash, seat, pour, spray, beckon, row, yellow, gather, die, glance, paint, crouch, color, stretch, flog, slit, affix, detect, install, spread, man, train, rush, adjust, function, smell, water, clean, discard, originate, own, cause, chew, snog, share, cuddle, jab, cancel, steer, teach, shake, dock, overcharge, name, anchor, foot, total, carve, advertise, suspend, buy, distribute, squat, smoke, milk, balance, shoot, blush, sew, photograph, lean, assemble, seize, tuck, lock, abscond, paddle, add, deprive, lower, emerge, let, drip, dress, include, dangle, urge, shop, print, forget, power, rub, litter, supply, bloom, swim, people, clock, guard, inspect, analyze, juggle, pick, burn, store, clasp, peer, sport, postpone, curl, gesticulate, film, dirty, shield, arrange, rock, resemble, etch.
