# Supplementary material for: Quantifying the roles of visual, linguistic, and visual-linguistic complexity in noun and verb acquisition
Source: PLoS One. 2025 May 23;20(5):e0321973. doi: 10.1371/journal.pone.0321973 (PMC12101840; doi:10.1371/journal.pone.0321973)
Supplement: S3 Appendix — (PDF) [file pone.0321973.s006.pdf]

# S3 Appendix

## Words in the Visual Relationship Detection Dataset

**Nouns** man, woman, sky, sign, building, bus, table, shirt, pole, car, wall, boy, window, train, girl, street, tree, water, truck, boat, hat, hair, floor, grass, chair, light, person, pants, trees, road, fence, jacket, ground, umbrella, plate, motorcycle, door, clock, sidewalk, people, bag, glasses, snow, desk, bike, bench, laptop, tracks, windows, shoes, tower, helmet, counter.

**Verbs** on, has, above, wearing, behind, under, near, in, below, beside, holding, over, by, beneath, with, carrying, riding, inside, touching, against, covering, at, around, watching, using, contains, eating, pulling, between, outside, says, crossing, before, driving, along, cutting, swinging, facing, playing, of, casts, across, beyond, sees, flying, pushing, blocking, making, matches, hitting, surrounding, casting, showing.
