# Supplementary material for: Quantifying the roles of visual, linguistic, and visual-linguistic complexity in noun and verb acquisition
Source: PLoS One. 2025 May 23;20(5):e0321973. doi: 10.1371/journal.pone.0321973 (PMC12101840; doi:10.1371/journal.pone.0321973)
Supplement: S4 Appendix — (PDF) [file pone.0321973.s007.pdf]

S4 Appendix

Words in the Moments in Time Dataset

clapping, dropping, burying, covering, flooding, leaping, drinking, raining, stitching, spraying, twisting, coaching, submerging, breaking, boarding, running, destroying, competing, giggling, shoveling, chasing, flicking, pouring, hammering, carrying, surfing, pulling, squatting, crouching, tapping, skipping, washing, winking, queuing, locking, stopping, sneezing, flipping, sewing, clipping, working, rocking, asking, camping, plugging, pedaling, constructing, slipping, sweeping, screwing, shrugging, hitchhiking, cracking, scratching, trimming, selling, stirring, jumping, starting, clinging, socializing, picking, splashing, licking, kicking, sliding, filming, driving, handwriting, steering, filling, pressing, shouting, hiking, vacuuming, pointing, giving, diving, hugging, building, dining, floating, leaning, sailing, singing, playing, bubbling, joining, raising, sitting, drawing, rinsing, coughing, slicing, balancing, rafting, kneeling, dunking, brushing, crushing, watering, removing, tearing, imitating, teaching, cooking, reaching, studying, serving, bulldozing, shaking, discussing, dragging, gardening, performing, officiating, photographing, sowing, dripping, writing, clawing, bending, boxing, mopping, gripping, flowing, digging, tripping, cheering, buying, bicycling, feeding, emptying, unpacking, sketching, standing, weeding, stacking, drying, crying, spinning, frying, cutting, paying, eating, lecturing, dancing, boiling, peeling, wrapping, wetting, welding, putting, swinging, carving, walking, inflating, climbing, shredding, reading, sanding, frowning, closing, hunting, clearing, launching, packaging, fishing, spilling, leaking, knitting, boating, sprinkling, rolling, spitting, dipping, riding, chopping, extinguishing, applauding, calling, talking, snowing, shaving, marrying, rising, laughing, crawling, flying, assembling, injecting, landing, operating, packing, descending, falling, entering, pushing, sawing, smelling, overflowing, waking, barbecuing, skating, painting, drilling, tying, manicuring, plunging, grilling, pitching, towing, telephoning, crafting, knocking, storming, placing, turning, barking, opening, juggling, mowing, sniffing, interviewing, stomping, chewing, grooming, rowing, bowing, gambling, saluting, fueling, autographing, throwing, drenching, waving, signing, repairing, baking, smoking, skiing, drumming, blowing, cleaning, combing, spreading, racing, combusting, swimming, shopping, bouncing, dusting, stroking, snapping, biting, roaring, guarding, unloading, lifting, instructing, folding, measuring, whistling, exiting, stretching, taping, squinting, catching, draining, scrubbing, celebrating, jogging, bowling, resting, blocking, smiling, tattooing, erupting, howling, grinning, sprinting, hanging, planting, speaking, ascending, yawning, cramming, burning, wrestling, poking, tickling, exercising, loading, piloting, typing.
